# Assessment of Skeletal Age in Children with Unilateral Cleft Lip and Palate

**DOI:** 10.5005/jp-journals-10005-1209

**Published:** 2013-10-14

**Authors:** MS Ravi, S Ravikala

**Affiliations:** Professor, Department of Orthodontics, AB Shetty Memorial Dental Institute of Dental Sciences, Mangalore, Karnataka, India, e-mail: drmsravi@gmail.com; Postgraduate Resident, Department of Orthodontics, AB Shetty Memorial Institute of Dental Sciences, Mangalore, Karnataka, India

**Keywords:** Skeletal growth, Cervical vertebra, Unilateral cleft Lip and palate

## Abstract

**Objective:** The objective of the study was to assess the skeletal maturity in children with unilateral cleft lip and palate (UCLP) and to compare it with that of the noncleft children.

**Design and setting:** It is an institution based study conducted on randomly selected subjects visiting the hospital for consultation and treatment**.**

**Participants:** A total of 60 children with UCLP (25 boys and 35 girls) and 60 noncleft children (30 boys and 30 girls) with normal occlusion in the age group of 10 to 15 years participated in the study. They were classified as younger (10 to 13 years) and older (13 to 15 years) age group and the cervical vertebrae were assessed in lateral cephalograms using Hassel and Farman, modifications of Lamparski's criteria.

**Statistical analysis:** Data was analyzed using Fisher's exact test.

**Results:** Younger age group children with UCLP showed significant delay in skeletal maturation when compared with the noncleft children, whereas older age group children with UCLP showed a faster rate of skeletal maturation when compared with that of noncleft children but the difference was statistically not significant.

**Conclusion:** Children with UCLP exhibit delay in attaining skeletal maturation when compared to noncleft children. There is a delay in skeletal maturation at younger age but not in older age group of children with UCLP.

**How to cite this article:** Ravi MS, Ravikala S. Assessment of Skeletal Age in Children with Unilateral Cleft Lip and Palate. Int J Clin Pediatr Dent 2013;6(3):151-155.

## INTRODUCTION

Cleft lip and palate is the most common congenital anomaly that occurs in humans. In India, the incidence of cleft lip and or palate ranges from 0.25 to 1.56 per 1000 live births.^1^The cleft usually occurs when some factor or factors alter the normal growth and development of the lip, palate and maxilla. The knowledge about these factors will help us to understand the etiology of cleft and also to improve the treatment of cleft patients.^2^

The weight and length of children with cleft lip and palate and isolated cleft palate were lower when compared with the children having cleft lip only.^3-5^ Specifically, males with unilateral cleft lip and palate as well as those with isolated cleft palate were significantly shorter and thinner (reduced BMI) than normal.^6^

In addition to weight and length, radius length, knee width and length of the tibia were also found to be reduced in cleft lip and or cleft palate children.^7^

Many studies have found that in cleft individuals, maxilla and mandible are retrusive, the cranial base angle is flatter, upper facial height is reduced, shorter mandibular ramus and body length, an obtuse gonial angle and significant downward and backward rotation of the mandible.^8-10^

Since, certain growth differences were found in cleft individuals, knowing these growth differences will certainly be advantageous during diagnosis and treatment planning for the comprehensive management of the cleft individuals.^3,5^ Growth modification is one of the most important treatment goals in dentofacial orthopedics especially in cleft children. It has an advantage of correcting skeletal imbalances during growth period and there by influencing and promoting skeletal and dentoalveolar correction.^11^

Hence, this study is planned and designed with the objective of assessing the skeletal maturity in cleft children and to compare it with that of the noncleft children using cervical vertebra method.

## MATERIALS AND METHODS

In order to assess the skeletal growth in children with UCLP and compare it with that of noncleft children, 120 children in the age group of 10 to 15 years were selected for the study and were classified into two groups as follows.

*Group 1:* Sixty noncleft children (30 boys and 30 girls), with pleasing profile, normal jaw relationship without any facial asymmetry and with near normal dental occlusion were selected. None of them had undergone any surgical/ Orthodontic treatment. They were subgrouped as;

*Group 1A:* Children belonging to 10 to 13 years of age

- 1A_1_–15 boys
- 1A –15 girls

*Group 1B:* Children belonging to 13 to 15 years of age

- 1B_1_–15 boys
- 1B_2_–15 girls

*Group 2:* Sixty children (25 boys and 35 girls) with UCLP with no other associated defects were selected for the study.

*Group 2A:* Children belonging to 10 to 13 years of age

- 2A_1_–12 boys
- 2A_2_–18 girls

*Group 2B:* Children belonging to 13 to 15 years of age

- 2B_1_–13 boys
- 2B_2_–17 girls

**Fig. 1:**
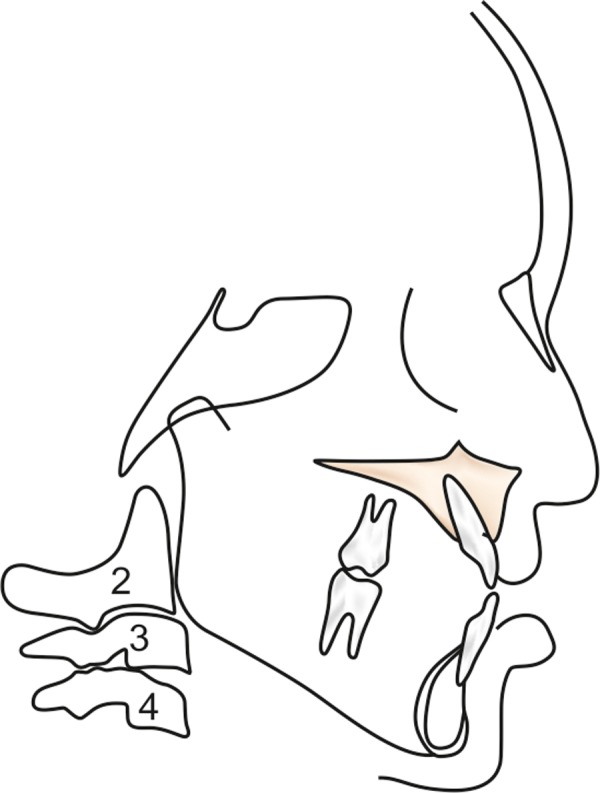
Lateral cephalogram tracing for cervical vertebrae assessment

### Method of Collection of Data

After obtaining the written informed consent, lateral cephalograms were made for each individual in a standard technique using Planmeca PM 2002 cc Proline (Planmeca, Helsinki, Finland) radiographic machine.

**Figs 2A to F:**
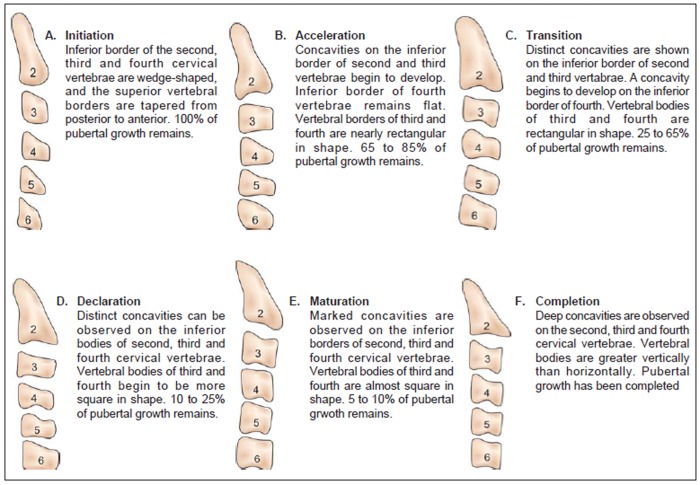
Cervical vertebra assessment as per Lamparski and modified by Hassel and Farman

Lateral cephalograms were traced for three parts of the cervical vertebrae (body of 2nd, 3rd and 4th cervical vertebrae) on 0.003-inch matte acetate with a 0.5 mm diameter lead pencil (Fig. 1). Cervical vertebra development of the sample was evaluated using Hassel and Farman, modifications of Lamparski's criteria (Figs 2A to F).^12^

Fisher's exact test was used to test the significance (p = 0.05 or less) between the groups.

## RESULTS

### 10 to 13 Years Age Group

In this age group, UCLP children showed statistically significant delay in skeletal maturation when compared with the noncleft children (Table 1). While both boys and girls with UCLP showed delay in skeletal maturation, the difference was significant only in boys with UCLP when compared to noncleft boys (Tables 2 to 4).

**Table 1:** **Table 1:** Comparison of skeletal maturation between cleft and noncleft children (10-13 years)

*Maturation stage*	*Count % within maturation stage*			*Fishers exact test*	*p-value*
	*Noncleft*	*Cleft*	*Total*		
Initiation	1	3	4	9.357	0.019
	25.0%	75.0%	100.0%		
Acceleration	6	10	16		
	37.5%	62.5%	100.0%		
Transition	17	6	23		
	73.9%	26.1%	100.0%		
Declaration	7	1	8		
	87.5%	12.5%	100.0%		
Total	31	20	51		
	60.8%	39.2%	100.0%		

**Table 2:** **Table 2:** Comparison of skeletal maturation between cleft boys and noncleft boys (10-13 years)

*Maturation stage*	*Count % within maturation stage*			*Fishers exact test*	*p-value*
	*Noncleft*	*Cleft*	*Total*		
Initiation	0	3	3	7.444	0.023
	0%	100.0%	100.0%		
Acceleration	4	2	6		
	66.7%	33.3%	100.0%		
Transition	11	3	14		
	78.6%	21.4%	100.0%		
Declaration	0	1	1		
	0%	100.0%	100.0%		
Total	15	9	24		
	62.5%	37.5%	100.0%		

**Table 3:** **Table 3:** Comparison of skeletal maturation between cleft girls and noncleft girls (10-13 years)

*Maturation stage*	*Count % within maturation stage*			*Fishers exact test*	*p-value*
	*Noncleft*	*Cleft*	*Total*		
Initiation	1	0	1	11.892	0.003
	100.0%	0%	100.0%		
Acceleration	2	8	10		
	20.0%	80.0%	100.0%		
Transition	6	3	9		
	66.7%	33.3%	100.0%		
Declaration	7	0	7		
	100.0%	0%	100.0%		
Total	16	11	27		
	59.3%	40.7%	100.0%		

### 13 to 15 Years Age Group

A faster rate of skeletal maturation was observed among children with UCLP when compared to noncleft children but the difference was statistically not significant (Table 5). Both boys and girls with UCLP showed increased skeletal maturation when compared to noncleft boys and girls respectively. However, the differences were statistically not significant (Tables 6 to 8).

**Table 4:** **Table 4:** Comparison of skeletal maturation between cleft boys and cleft girls (10-13 years)

*Maturation stage*	*Count % within maturation stage*			*Fishers exact test*	*p-value*
	*Boys*	*Girls*	*Total*		
Initiation	3	0	3	6.936	0.046
	100.0%	0%	100.0%		
Acceleration	2	8	10		
	20.0%	80.0%	100.0%		
Transition	3	3	6		
	50.0%	50.0%	100.0%		
Declaration	1	0	1		
	100.0%	0%	100.0%		
Total	9	11	20		
	45.0%	55.0%	100.0%		

**Table 5:** **Table 5:** Comparison of skeletal maturation between cleft and noncleft children (13-15 years)

*Maturation stage*	*Count % within maturation stage*			*Fishers exact test*	*p-value*
	*Noncleft*	*Cleft*	*Total*		
Initiation	0	2	2	4.782	0.277
	0%	100.0%	100.0%		
Acceleration	8	15	23		
	34.8%	65.2%	100.0%		
Transition	10	15	25		
	40.0%	60.0%	100.0%		
Declaration	11	8	19		
	57.9%	42.1%	100.0%		
Completion	1	0	1		
	100.0%	0%	100.0%		
Total	30	40	70		
	42.9%	57.1%	100.0%		

**Table 6:** **Table 6:** Comparison of skeletal maturation between cleft boys and non-cleft boys (13-15 years)

*Maturation stage*	*Count % within maturation stage*			*Fishers exact test*	*p-value*
	*Noncleft*	*Cleft*	*Total*		
Initiation	0	2	2	1.832	0.700
	0%	100.0%	100.0%		
Acceleration	7	8	15		
	46.7%	53.3%	100.0%		
Transition	6	8	14		
	42.9%	57.1%	100.0%		
Declaration	4	3	7		
	57.1%	42.9%	100.0%		
Total	17	21	38		
	44.7%	55.3%	100.0%		

## DISCUSSION

Clefts of the lip and/or palate are multifactorial in origin. The cleft results in a number of oral health and medical problems among the affected children. The very nature of the cleft raises apprehensions about feeding difficulties, which can lead to failure to gain weight and hinder proper growth and development.^1^

**Table 7:** **Table 7:** Comparison of skeletal maturation between cleft girls and noncleft girls (13-15 years)

*Maturation stage*	*Count % within maturation stage*			*Fishers exact test*	*p-value*
	*Noncleft*	*Cleft*	*Total*		
Initiation	1	7	8	5.482	0.106
	12.5%	87.5%	100.0%		
Acceleration	4	7	11		
	36.4%	63.6%	100.0%		
Transition	7	5	12		
	58.3%	41.7%	100.0%		
Declaration	1	0	1		
	100.0%	0%	100.0%		
Total	13	19	32		
	40.6%	59.4%	100.0%		

**Table 8:** **Table 8:** Comparison of skeletal maturation between cleft boys and cleft girls (13-15 years)

*Maturation stage*	*Count % within maturation stage*			*Fishers exact test*	*p-value*
	*Boys*	*Girl*	*Total*		
Initiation	2	0	2	2.225	0.675
	100.0%	0%	100.0%		
Acceleration	8	7	15		
	53.3%	46.7%	100.0%		
Transition	8	7	15		
	53.3%	46.7%	100.0%		
Declaration	3	5	8		
	37.5%	62.5%	100.0%		
Total	21	19	40		
	52.5%	47.5%	100.0%		

Some authors have found lower mean birth weights in infants with cleft lip and/or cleft palate.^11,13^ Few other authors found no differences in birth weights between children with cleft and without cleft.^3,14,15^

Few investigators suggested full recovery of perinatal weight loss by 6 months of age,^14^ where as others suggested that this recovery takes place over period of years rather than months.^4^

This temporary growth lag is associated with the severity of the cleft and could be due to early feeding difficulties, a tendency to frequent upper respiratory infections, intestinal disorders and repeated hospitalization for lip and/or palate surgery.^4,5,16^

Some investigators observed both the height and weight of the cleft children to fall below that of the noncleft children after 10 years of age. They suggested that this diminution resulted from events in adolescence (endocrine controls of maturation at puberty) and not the result of feeding difficulties, infections, or surgical interventions experienced in the months immediately following birth.^16^

It was noted that the males with cleft lip and palate have a less noticeable growth spurt during puberty and their total growth period was longer. Thus, were able to catch up with the normal control group. It was concluded that a growth hormone deficiency is not the likely cause of the growth retardation.^4^ This reveals the diverse and contradictory nature of the morphologic variation observed among patients with clefts.

Hence, this study is designed with objective of assessing skeletal growth in children with UCLP using cervical vertebra method and to compare it with that of the noncleft children. 60 children with UCLP in the age group of 10 to 15 years were selected. These groups of children were further classified in to a younger age group (10-13 years) and an older age group (13-15 years) for the convenience of skeletal maturity assessment. Their vertebral developmental status was assessed using the Hassel and Farman, modifications of Lamparski's criteria and was compared genderwise with that of 60 noncleft children in the same age group.

In the present study, the children with UCLP of 10 to 13 years age group irrespective of gender showed delay in skeletal maturation when compared with the noncleft children.

A study by Ross RB reported that the skeletal age is retarded in cleft children and the cleft children are shorter and lighter than that of control children. The authors suggested the reason for height-weight retardation to be due to feeding problems and heightened frequency of infections.^9^

Hunter and Dijkman reported that between age 3 and 10 years, the affected twin was not shorter or lighter than his or her twin. After 10 years of age, both height and weight of cleft twin tended to fall below the normal twin.^17^

Duncan et al also reported difference among the growth pattern of children with cleft, wherein they found that isolated cleft palate children showed growth pattern which nearly simulates that of patients with isolated growth hormone deficiency.^18^

These findings were different from a study by Ranalli and Mazaheri^19^ who reported that in general, cleft groups do not age-for-age and sex-for-sex show any real departure from the noncleft averages. However, the authors suggest that children with clefts experience a growth lag following birth but that by 3 years of age they have caught up with the normal.

Jensen and Krieborg et al^4^ evaluated skeletal maturation by measuring mean height, radius length, knee width and length of the tibia in patients with cleft and compared it with the normal controls and reported significant difference between the groups The authors also observed that cleft palate males continued to be smaller than unaffected males through adolescence, with significant difference seen at 8.5, 9.5 and 10.5 years of age.

Rudman and Davis et al^20^ found that heights of cleft children to be below the 3rd percentile for that age group and suggests that children with cleft lip or cleft palate are

40 times more likely to experience growth hormone deficiency than the noncleft children.

In the present study, girls with UCLP of 10 to 13 years age attain skeletal maturation faster than cleft boys of similar age. Similar significant difference in cleft boys was reported by Sun and Li^21^ where the authors observed that boys with cleft lip and or palate were at a higher risk of delayed growth period and retarded pubertal growth peak.

Bowers and Rosario et al^6^ found that specifically males with unilateral cleft lip and palate and isolated cleft palate were significantly shorter and thinner (reduced Body Mass Index) than normal, where as females with isolated cleft palate differed from normal only in their shorter height.

The present study showed that children with UCLP of 13 to 15 years of age group, irrespective of the gender showed faster rate of skeletal maturation than noncleft children, but the difference was statistically insignificant.

The findings of the study suggest that in 10 to 13 years age group there is delay in skeletal maturation among children with UCLP; where as in 13 to 15 years age group skeletal maturation was found to be comparable among children with UCLP and noncleft children. Possible reason could be because of the adolescent catch-up growth^6^ seen among the cleft children. The total growth period is longer among cleft children and thus they are able to catch up with the normal control group.^4^

## CONCLUSION

Children with UCLP exhibit delay in attaining skeletal maturation when compared to noncleft children. The result of the present study shows that there is a delay in skeletal maturation at younger age but not in older age group of children with UCLP. In order to provide comprehensive care with respect to each patient, this factor needs to be considered during the diagnosis and treatment planning for the children with UCLP.

Further studies are required to assess the skeletal growth in different types of orofacial clefts and in different ethnic groups.
